# On Civilization and Insanity

**Published:** 1857-04-01

**Authors:** F. Parigot

**Affiliations:** Formerly Inspecting Physician to Gheel; Member of the Committee of Inspection of the Establishments for the Insane in Belgium. (Written expressly for this Journal.)


### Art. VII.—ON CIVILIZATION AND INSANITY.

BY DR. F. PARIGOT,

Formerly Inspecting Physician to Gheel; Member of the Committee of Inspection of the Establishments for the Insane in Belgium. (Written expressly for this Journal.)

True civilization is that which results from intellectual progress and the practice of morality in every class of society. The degree of science, and even of charity, with which the insane are treated in every country is in proportion to the degree of civilization such as we have just defined it.

But civilization may be, as certain pessimist writers pretend, the very cause that develops this disease in our time. We do not believe it. Professing this opinion, it remains for us to develop, in this memoir, why statistics show, during the last ten years, an increase in the number of the insane ; why we, on the contrary, believe that, in proportion as true civilization shall advance, insanity will diminish its ravages in society ; and, lastly, amongst the means to be opposed to this disease we will mention the treatment by *free air* as the best. We proceed, therefore, to explain our ideas and our convictions upon this subject. They are based upon the observation of facts ; the imprescriptible rights of the insane ; the duties towards them which are incumbent upon us ; and, lastly, on the various methods which the insane have been made to undergo in those countries where it is assumed that civilization is most advanced.

No one can deny that, the more a people is advanced the more will its attention be directed towards the means which education and physiology supply, to lessen, as far as practicable, the causes of a disease so grave as to destroy reason, even for a moment. It cannot be doubted that in this direction such a people would strive to combat the results, especially if it thought that the evil was spreading in the different classes of society. Well, any government wishing to accomplish this important end would naturally be led to encourage the study of a science which treats of the diseases of the mind—diseases which might well be more frequent at epochs like ours, when everything is bent towards personal gratifications. Such a government would therefore teach psychological medicine in its schools, in order that the new generation of physicians should possess at least the first principles ; it would encourage the attempts or innovations which this science might initiate either to cure the curable insane, or to ameliorate the condition of the incurable by restoring them to society, by admitting them to the domestic fireside, and especially by avoiding the construction of those vast and costly agglomerations of lunatics, in which dementia seems in the end to establish a permanent reign.

All this would certainly furnish for a model-people the sufficient reason to modify what had been recognised to be defective in any system of treating the insane. Unhappily, we should seek in vain amongst republics or monarchies for this people wise and foreseeing, which exists no more.

The history of humanity, unlike that civilization for which we hope, shows us the reverse of this in ancient and modern times, if, indeed, we except fifty years.

The state of barbarism of nations, and the coarseness of their manners, have had for their consequence the negation of all pity for those who suffer at the same time both in their moral and physical constitution. It may be said that in ancient times, and even now, there has not been, and is not yet, any perfect knowledge or true practice of the duty owing to the insane, as it ought to be understood, without calculation and without any hope of reward. Moreover, we may behold ancient peoples, when they were organizing themselves—formed for the most part (as we see in North America) of elements collected by chance,—seeking at first to free themselves from everything that importunes them, and which seems to hamper them in their course : they have few hospitals and prisons ; they are useless, for, most frequently, they hang their criminals without trial ; and woe to him who needs public aid, for he is abandoned to his lot. Our ancestors, too, had little pity for those who could not follow them to battle. Ransack history : the insane are objects of buffoonery, or burnt as being convicted of sorcery. Later—they are abandoned, and they perish for want of care in hideous prisons. At the present day, even, do they not execute maniacs before the eyes of all the world, in spite of the protestation of science ? When all these things are considered, it is not surprising to me that on the Continent the insane are held within the jurisdiction of the Minister of Justice, but in the division of the condemned.

Each people has its own manner of seeing things. Excepting the Turks, who still maltreat their insane, the Orientals add a certain derision to the want of care. They look upon the insane as inspired, and thus, like ourselves when we want to drive away a beggar, they abandon them to the *grace of God!* Fortunately, there public pity and liberty are not denied them. If this is what some uncultivated and fanatic nations still do, what is then the charitable practice of the civilized West? It is this: our first feeling at the sight of a sick man who is delirious is that of fear and disgust, and our instant resolution is to put this object of dread out of sight. For the poor there exist closed asylums, which too often fulfil the purpose of the ancient *Oubliettes.* This is, at least, the idea formed of these establishments by the public. But Government, which has considerably improved them, urges the erection of closed asylums in every province, because thus vagrancy is rendered impossible. It was in this spirit that the French law of the 24th August, 1790, was framed. This provides for the avoidance of the disastrous events occasioned by *furious* lunatics left at liberty, and against the wandering of *mischievous* and *ferocious* beasts! Singular combination! This law ordered cells for the first, and cages for the last! Behold what prejudice ordained at the moment of a reform which ruined so many abuses. These prejudices permitted the confusion of men and brutes, and made no distinction between the different kinds of insanity, although it is easy to see that nine-tenths of the insane offer no kind of danger to the common life. As for the rich insane, or those belonging to the middle classes, speculation arose to meet the necessity of removing the patient, as well as that of ridding oneself of a turbulent guest. Capitalists, highly honourable, I am happy to say it, have calculated that for a certain investment it was possible to entertain suitably and economically a large number of insane patients at the same time, and thus to draw considerable profits from an industry which, after all, is not of the most agreeable. Competition has even led to the reduction of the charges, whilst the dearness of provisions has still further diminished the profits. Thus, we are acquainted with one of these speculators, worthy of sympathy, who has preferred encountering losses rather than to lessen the comforts of his patients.

In general, these are the tendencies of our age: to ameliorate the condition, material and moral, of the secluded, but at any rate to keep them secluded.

We propose to retrace the ameliorations, and to mention the efforts of the men who have recently striven to lead public opinion to better sentiments, and especially to restore to science the position which it becomes her to occupy in the treatment of mental alienation. We cannot conceal from ourselves that hitherto the scientific element has counted but for little in nearly all public or private establishments, unless it be those governed by eminent medical men. Some distinguished men have exclaimed against this state of things; but then they are, according to the world, Utopians, or physicians called *Narren*-doctor, *Zotten*-doctor, or *Mad*-doctor. As a rule, administrative authorities and speculators trouble themselves little about psychological physicians; and what an ignorant public imposes upon them in the way of privations, they are compelled to carry out, to the detriment of the insane.

The following dialogue will lift up a corner of the curtain:—

*A Relative* (to the Superintendent Proprietor).—Sir, I understand that you have different prices for patients?

*The Proprietor.*—Yes, Sir; but the care and the attention paid are the same for all my inmates, excepting as to the room, the number of dishes, and certain extras which depend upon the scale of payment.

*The Relative.*—Well, as my patient scarcely eats at all, I make up my mind for the lowest scale. Besides, you tell me the care is the same. You will be kind enough to give him all the *extras* for which he may ask. We should be so pleased to have him well treated!

*The Proprietor.*—Let us understand one another; for experience has taught me that these extras are often useless when they are paid for separately.

*The Relative.*—That is, give him all that is *indispensably* necessary. Well, we beg of you to let him see no one; this point is very important. Our misfortune must not be known. On this account we have even been on the point of sending him abroad.

*The Proprietor.*—Will you employ a specialist physician? This would be at your cost.

*The Relative.*—No, no! it costs us enough as it is.

After all, at the worst, what could happen if science were entirely put aside? The patient would have but little chance of recovery; or he would soon die; or, lastly, he would fall into the chronic state—into that twilight of reason in which the life of a lunatic may be prolonged in the silence of a cloister, or of a division of "*quiet* patients." There are proprietors who have even written and maintained that there was no mental medicine, and that sojourn in their asylums answered every want! Truly, it seems hardly worth while to disturb a state of things so agreeable—with which everybody seems satisfied—except the lunatic.* But what, after all, is this unfortunate? If he returns into the world, cured, he has lost the consideration in which he was held; he is exposed to slander; and if he is slow in recovering, ought he not to think himself happy in being allowed to live amongst his fellows? It is only a madman, a wretch who has lost all—rank, family, and even fortune, which is sometimes inherited whilst he still lives. Clearly it only remains for him to create for himself a new existence—a fictitious life like that of his companions of the insane, and a few servants more or less pleasant. He will remain, as has been said by our respected friend, Dr. Biffi, of Milan, for long months, for long years, in the same place; a hundred times a day he will see the same objects and the same scene, bounded by the inexorable wall of the enclosure. At this price the finest apartments become unendurable, and the most beautiful gardens put on a frightful monotony. Better a thousand times the last hovel of the labourer in the open country. Fortunately there are exceptions to this cloistral *régime;* there are also institutions where this weary tedium is diminished; but these are exclusively for the rich, and are directed generally by the most eminent men in the medical profession, whose reputation has attracted patients of this order. And even these houses, if they admit too large a number of patients, necessarily lose the particular character and the kind of care which it belongs exclusively to the family to impart.

We cannot help saying that, if in our time a certain stage of amelioration has been reached which some persons look upon as the ideal of what can be accomplished, we have, nevertheless, entered into a path quite artificial and pernicious in a therapeutic point of view—that is, of erecting everywhere vast phalausteria of lunatics, in which the public may rid itself, at a cheap rate, of beings irksome to society. From the prejudice mentioned above often proceeds the denial of justice and of medical care. There is refused generally: — first, the most suitable means, and often all means, of cure; secondly, domestic life; thirdly, relative liberty; fourthly, the enjoyment of private fortune in its application to personal wants;—all this because mental alienation is still regarded as a stain that only oblivion or death can wipe out. This is why it has been said with truth, that it is better to be dead than to be struck with one of those numerous affections, of which the whole is called insanity.

What is curious enough is, that the great nations have each of them, more or less, pretensions to a certain superiority in power, riches, science, and some morality, and that it is to this latent

* It is not necessary to say that there are exceptions to this rule, and that they are the more honourable because they are rare.

relation between the degree of civilization and of beneficence, that we must attribute the efforts of certain German, English, and French alienists to raise the moral worth of their country by competitive boastings of the ameliorations that have been realized in each. This competition is good ; but we shall have occasion to remark how imperfect these ameliorations still are, and especially how badly they are directed, because they end in nothing but the satisfaction of spending enormous sums in the erection of immense palaces, where it appears that infirm populations, continually renewed, will come, to perpetuate their sad condition.

Yes, notwithstanding the progress of enlightenment, and the good which it produces, it will yet be a long time before the effects will reach the insane through the obstacles heaped up around them by time and prejudice. Instead of the self-gratulations of the time, would it not be more just that the history of the bad treatment which the insane have had to undergo, and which they still have to undergo, by far outweighs the catalogue of boasted ameliorations. That long list of torments formerly inflicted as punishments, and now, under the name of *moral treatment*, contains a series of facts, of which the odious and ridiculous aspect has been perfectly appreciated by Dr. Ramaer of Zutphen, in Holland, who has made this the subject of an excellent article in the *Nederlandsch tydschrift voor psychiatrie.*

Besides, it is not so long ago that these ameliorations have been introduced ; and it is matter for astonishment that it is almost at the end of the nineteenth century, that the nature of our affective sentiments, and especially that Christian morality, had at last reacted upon our institutions as regards the insane. For the honour of the medical profession, this path has been opened by physicians. In different countries, it is to Pinel, Daquin, Samuel Tuke, Langerman, Ferrus, Chiarurgi, and in our country to Guislain, that we owe the reform of the government of the insane. It belongs, however, to all the world to follow in the steps of these beneficent men—to advance in the path of progress ; for all is not yet accomplished in the way of restitution to men who for a long time have had no voice that could be heard in the world.

Recently, some well-meaning persons have believed, when they beheld religious congregations devoting themselves to the establishment of asylums for the insane, that thus a great good and progress would be achieved. But it must be said, because it is the truth, that with the exception of the incurables and idiots, this is an error. The man of the world, the mother of a family, young people, suddenly seized with this disease, can these derive any benefit from a medium in which nothing recals to them what they have lost? — a medium whose idea, whose metaphysical aims, they are for the most part unable to understand, and in which the wants and the aspirations of actual life are most commonly ignored. How much time, and how many mutual concessions are necessary in these asylums, where such a variety of natures are assembled! No: we sincerely believe that convent-life is the very worst for the insane as regards their recovery. We maintain, upon ample experience, that anywhere than here religious ideas will have more influence, and will be better received. Besides, it is obvious that the future comfort of the insane only depends upon the manner in which each of us shall comprehend his duties towards them, and very little indeed upon the moral compression and the physical continence that can be exercised over them.

Thus, what nature and charity exact, actual civilization cannot yet give; for it does not admit the rights of the insane, or the obligations which these impose upon us. Our epoch is therefore but one of transition, during which, if we seek to do good, we must not fear to deviate from the boundaries that private interests and the ruling égoism might counsel us to respect. Let us then tell the truth: the reform of the government of the insane is far from being capable of being arrested; it is yet but at the beginning of its work; the public conscience has not yet spoken; and certain learned men, still in error, seek only for means of restraint to obtain the cure of insanity; whilst gentleness, patience, a country life, and the resources of medicine might suffice. Actually, as Dr. Ramaer proves, we have substituted for the old methods of violence, others as cruel, but analogous to the first. Now, without wishing in this article to enter into therapeutics, we may say, that the question for science to determine is, whether, yes or no, isolation shall be accompanied by physical and moral restraint?

In the rapid review of the practice of different countries, we shall have the opportunity of comparing the methods employed with that followed at Gheel, a Belgian village. We shall prove how the free air of the country, the contemplation of nature, agricultural labour, and especially family life enjoyed amongst good and simple country-folk, constitute a moral and natural medication more consistent with true civilization than any other. Peasants perform through zeal, through the traditions of example, through necessity, perhaps, what we ought to do through duty, and conviction of the resulting benefits. Let us hope then that the future will solve what still causes the insane to be excluded from the circle of humanity.

According to Reid, it was towards 1803 that the barbarism of ancient times began to lose its intensity in Germany; before this epoch, he says, we shut up the insane like malefactors, in the cells discovered in old dilapidated prisons, which had long become the habitual dwelling of owls ; or else, in order not to hear them, we hid them in the belfries of the communal towers, or in the cellars of houses of correction, wherever, in short, no look of compassion could find them. At the present day, adds Mr. Ramaer, in the memoir above cited, there exists in Germany no public establishment whose regulations forbid the abolition of methods of violent repression, and in which, on the other hand, we do not find the prejudice in favour of their necessity rooted in the mind of the superintendent. The strong arm-chair, the rotatory apparatus, the bonds and fetters of every kind, douches, strait-waistcoats, &c., constitute the basis of the penal code in force. Lastly, many of these establishments are of a mixed kind ; that is, appropriated of the reception of correctional delinquents as well as of the insane.

In this country philosophical and religious opinions have not been without influence on the condition of the insane. Thus, when transcendental metaphysics reigned in its schools, the question of first causes, and of their influence upon the human mind, was discussed ; it was then that Dr. Heinroth—a learned physician, be it admitted—pretended that mental diseases drew their principle from the community between the human soul and the Devil ; which, according to him, admirably explained why the insane are neither free nor reasonable. He believed himself thus authorised to deliver his patients from this possession, and for this purpose he employed correctional wards, furnished with all the machines and engines necessary for this expurgation. He recommended that in each ward should be placed *four executioners* for two hundred lunatics. Another physician, Lichtenberg, pretended that blows with a stick was equally useful in the treatment of insanity ; because the soul is then forced to cling firmly to the positive world. Lastly, a certain Dr. Picht asserted that two or three blows with a birch stick worked wonders in the cure of insanity.

At the present day all Germany condemns these cruelties ; and nothing that we can say can equal the force with which this system is repelled by alienist physicians. As to those abstract discussions upon the question of knowing if insanity depends upon a state of sin,—if the soul can be sick,—if the body and the soul can be in relation with spirits or the Devil,—all these have vanished from amongst psychiatric physicians worthy of the name, because they are more capable of probing the wounds produced either by the brutality of materialism, as well as the insanity and the perversion of a mysticism that only seeks to traffic upon humanity.

In this vast empire the first reforms were effected by the celebrated Langerman; they first penetrated the asylum of Sonnenstein, in Saxony. However, it is only since 1820 that new asylums, built according to the rules of art and the ideas of the day (which exact a crowd of compartments and divisions—the despair of architects and classifying physicians)—it was only then that these asylums were erected at Schleswig, Siegburg, Heidelberg, Prague, Vienna, &c. The patients were divided into two categories, the curable and incurable; lastly, in order to secure method and tranquillity, they were further subdivided into *violent*, *quiet*, *half-quiet*, *dirty*, *idiots*, and *convalescents*. Thousands of plans were contrived to discover the $x$ of a good classification. Every alienist physician of any repute must even travel a long time, and visit the principal asylums of Europe to detect the indications in all the imaginable forms of straight lines and curves, but he will take especial care not to visit Gheel, where the difficulty is completely overcome. However, the establishments which we have just cited are remarkable for their excellent organization; they contain schools, libraries, gymnasia, recreationary wards, &c.

In general, German physicians practise but sparingly a system imagined some years ago in England, by which all restraint was abolished,—called in consequence, the "non-restraint" system.* They pretend that this is an Utopia in an enclosed establishment, and that no discipline is possible without material means of repression; not to speak of cases of acute mania and of violent dementia, in which restraint is indispensable. It appears, indeed, difficult to suppose that, in a large assemblage of patients, idiots and epileptics, all more or less irritated, from being confined, it is possible to establish order by means of exhortations, or by appealing to the affections of maniacs. However this may be, all writers in our day are agreed in declaring that Germany is the model country for medical treatment (?). We find there registers in which are recorded the history of the affections of each patient—the symptoms, diagnosis, prognosis; the prescriptions, and daily visits of the physicians are so recorded, that the recovery or autopsy come, so to speak, to approve or condemn the methods pursued. It is plain that in such a manner science may make true progress, and that the patient derives a real benefit. What an interval between this broad spirit of examination and appreciation of things and those conditions, paltry and discreditable, which are sometimes associated with the functions of alienist physicians in certain countries! Who would believe that there exist establishments in which the physi-

* This term indicates the negation of an evil. *Free air* is the affirmation of a good.

cian is required to take charge of two or three hundred patients for five or six hundred francs a year! In Germany, there is for every ayslum an adequate number of physicians to allow each of them to institute a conscientious examination of the patients, to study the new cases, and lastly, to keep themselves on a level with the science they are called upon to practise. We are convinced that the result is material and moral benefit for asylums honourably conducted.

From what we have just related, it follows that in Germany, as everywhere else, the insane were for a long time abandoned, or, at least, much neglected. From this fact it may be concluded that the development of civilization has been long and slow; and also that it will not stop in the path of ameliorations and their consequences. No country can count a greater number of learned psychologists, such as Ideler, Bergman, Damerow, Guisenger, Fleming, Roller, Tessen, Nasse, Jacobi, Droste, Laehr, Erlenmeyer, Eulenberg, &c. &c. Private asylums have been established for the easy classes, and in these, we may be assured, the family life is perfectly applied to the treatment of insanity; we find there united all the cares and luxuries that lessen pain and restraint; such as the asylums of MM. Tessen, at Hornheim, of MM. Engelken, at Obernenland, and at Rockwinkel, near Bremen; of M. Erlenmeyer, at Bendorf, near Coblentz, &c.

If, then, the insane have had to wait a long time for ameliorations, they now possess them in as complete a form as the current ideas will allow. Why did we need so many centuries for this? Must this delay be attributed to the ancient rudeness of the Northern people,—to the simplicity of their manners? It is perhaps difficult to say, but this is how we understand it: during the long series of years that peoples take to settle themselves in a country, during what is called their youth, in the midst of wars or great works, which assure for them a material and political existence, alienation is rarely produced; movement seems to carry off and destroy the germ of this disease; it is only after a long time that man has been able to apply himself to the cultivation of his intellect, of his destiny, of the arts and sciences; and then it appears. New wants then arise; the external combat, from material that it was, becomes internal and moral; suffering arrives, and long years elapse before the abuses of the first institutions, founded upon force and the right of conquest, are replaced by those which have for their foundation reason and duty. This eternal struggle of the mind and matter then begins, in the order and value of political and religious institutions. Then the time comes when minds are exalted; then the intellect is exhausted, the passions are excited, and all this often in relation with sufferings which have induced a degeneration of the organism, of the temperaments and of manners. Then insanity breaks out; but can it be said that civilization is the cause? No; it is a period of struggling that we must pass through; it extends to the interests of humanity. It even happens that the system of public and private education has become so imperfect that it no longer answers to the real wants, and only serves to prepare deceptions for those who enter the different careers open to the activity of man. Yes; these times; are times of transition, which bring evils that civilization will destroy. In result, it appears that the number of lunatics has much increased; but let us compare epochs with each other, and we shall have the conviction that insanity is, after all, only in proportion with a more numerous population more instructed, and which demands far other things than contented it in former times; then also this disease was rare,—it remained ignored, and received aid from no one.

Let us open history, and we shall see that the same nature, the same country is not always able to oppose the same resistance to morbific causes, physical or moral. Nations traverse phases of development and of decay. Who shall say, therefore, that the civilization of humanity does not advance? We believe that causes may act unfavourably on certain countries, especially when the ferments of the future appear to be everywhere in action, without accusing civilization.

As to the treatment of insanity in Germany, it was null for centuries. Griesinger, the learned Professor of Tubingen, says in one of his works, that once within the walls of one of the prisons devoted to lunatics, a man never came out again. For more than thirty years this state of things has ceased; and, if it is later that in other countries the care of these diseases has been undertaken, this arises from the fact that with this people, so profoundly reflective, ideas had outstripped the means of executing reforms.

As we have said, physicians were the first who effected reform; we do not, however, assert that the initiative entirely depended upon them, or that reform was solely due to their zeal. It must be admitted that, in their epoch, reform existed in the ideas of the mass. Every one knows that at the end of the last century nearly all institutions were regenerated; at this epoch of investigation and of labour, a question so important as that of the security of personal liberty could not be overlooked. Everything was examined; and a generous physician, the celebrated Pinel, aided by a man not less meritorious, the administrator Poussin, profited by the opportunity to ameliorate the lot of those upon whom a cloistral life was imposed. The great principle of division of labour had just been recognised as indispensable in medical science, and it came to pass that the splendid specialities we have cited above were soon brought forth. Then insanity, that abject disease, until this period attaching itself to that new science which these men had created, ended by acquiring an importance of its own. In reflecting well, it was suspected that it was possible to fall into insanity very honourably. In fact, the amelioration of the lot of nations often depends upon individual efforts; but it had hitherto been forgotten. How costly, then, these efforts had been to those predestined workers in the progress of science and the arts. No one had related how many of these men had lost their reason in this gigantic labour—not through pride, as we hear the vulgar say, but as the consequence of their exertions, and often because they had to undergo the deceptions of their contemporaries. How many statues have since been erected to celebrated madmen! Byron, convinced of this fact, asserted that the less of folly the less from *above*. Well, civilization still wants devoted men. Genius will still be unappreciated; excellent inventions will still lead to misery and sickness! Are we to say, for this, that we must remain where we are, and then fall back?

It is intelligible that in their first application, the Good, the True, Liberty, may have been the cause of the loss of a crowd of men; but Evil, under the shapes of ignorance and despotism, have destroyed many more, and without any compensation whatever. Progress, in her march, tries every method. The legitimate desire of attaining truth has led to miscalculations; but it is no reason why human activity should stop itself, because some men faint under the load! Happy those who prove useful to humanity; their mission has been fulfilled. But, after all, this loss is but of trifling moment when compared with the advantages that the future will gain from it. One day the good will preponderate. It is in necessity that all sciences take their rise; it is for good that all the means of Providence are legitimate, although we cannot always follow the windings she takes to reach her goal. Thus, to return to our subject. Psychiatry was born, and must serve to combat those diseases which, according to certain persons, are augmenting with our tending to civilization—that is, towards good. It is at least a consolation in these difficulties to think that it will yield us the secret that will dry up the source of these diseases.

It is interesting to study what a great nation like France has done for the cause of the insane, since the epoch of Pinel whilst following her course towards civilization.

Very distinguished French alienists have published many works and memoirs on insanity. They have also described with care the principal public establishments of Paris and the departments. Many of these last are, according to these reports, improved, and have become in a measure model asylums; but they are not numerous or extensive enough to receive all the sick. In this respect the French law of 1839 has remained a dead letter. Colonies of lunatics would therefore be a blessing.

Here we must clearly understand the meaning of the word civilization; for France has always arrogated to herself the most advanced civilization, and the belief in her own intellectual and social pre-eminence has never ceased to prevail. One of her celebrities, M. Guizot, has indeed affirmed in his Lectures, that this pretension is philosophically legitimate. Every people, he says, recognizes the charm of the social relations, the gentleness of manners, the easy life, and at the same time the intellectual development of the French nation; no one disputes the qualities which distinguish Frenchmen: but is this the kind of civilization that is to better the lot of the insane? No; for it is easy to perceive in the works referred to above, that even in France the alienists are obliged to submit to every kind of opposition from the Administration, from its jealousy, its bad will, and especially from the neglect to which a crowd of lunatics are even yet consigned. Would it be believed, for example, that some unhappy beings are unable to reach the asylum common to four or five departments alive? Where is the recognition and the practice of duty here; and of what avail in such a case are the superiority and the charm of social relations?

The French Government ought to profit by the possession of so many earnest psychological physicians; and yet, read the programmes of her medical schools—you will not meet with a single course in which that instruction is officially given. As for the advantages of which M. Guizot speaks, they are really of little importance. Moreover, they no longer belong exclusively to any nation, and it seems to us inaccurate to pretend that in France the individual and society have equally progressed towards moral, intellectual, and material perfection. Every nation strives to reach this; let us hope that one day the common end will be attained, for it is on this triple condition that depends the diminution of our infirmities.

Chateaubriand has said that civilization does not describe a perfect circle, nor move in a straight line; it is, he says, on the earth like a ship at sea: beaten by the tempest, this ship makes her way, regains her course, falls sometimes below the point whence she set sail; but in the end, she meets with fair winds, and every day makes good something on her true course; and, lastly, makes the port for which she was bound. Let us not be deceived: display, luxury, the theatrical pomp of public ceremonies, are no certain signs of civilization and happiness; they rather hide a deep abyss when the moral element does not protect them. Truly, the material conditions of well-being are indispensable in order to bring forth the spiritual and moral germ implanted in us; but nothing must be exaggerated in what surrounds it, either in the physical or moral conditions; superfluity may injure as much as misery, which strangles the mind. How many false impressions (the source of many misfortunes, of insanity itself) have remained in the brains of those puppets that luxury delights to fashion!

Some authors have maintained that man in his natural state has only simple tastes, and few wants; but that from the moment that he civilizes himself, he acquires by this fact a mass of wants, engendering in their turn pains which may lead to folly. Is it then the want of the enjoyments and the riches of false civilization which makes insanity more frequent in our time? If that were true, we must despair of humanity; but this mode of reasoning seems to us false. If man only felt those wants to which his organs give rise, he might content himself, like animals, with the enjoyments which they give; but his intelligence, his feelings, his voluntary activity, revert to him the enjoyments of the mind and of the heart: those are the enjoyments he prefers. If he abuse his intellectual and moral faculties, if he exaggerate his organic instincts, if despotism brutalize him, he certainly creates for himself fictitious wants or shameful enjoyments, which will alter his sensibility, or give him a sickly exaltation. Happiness, as the vulgar understand it, blunts the feelings and the mind; and every day we see men exhausted ready to fall into melancholy.

We might then conclude, from what precedes, that amongst the causes which lead to insanity there are material and immaterial; that they may take their origin in the social and industrial relations, in the mode of instruction, in the religious relations, and lastly in moral error and organic degeneration. It is then the conscience or the organism, which, primarily attacked, react on the indissoluble link of mind and body. Thus, for example, misery vilifies the body in order to degrade the mind, whilst moral perversion kills the mind and heart first before destroying the brain by dementia.

France alone still possesses, as far as we know, the glory of having seen arise a medico-psychological society, composed of illustrious philosophers and physicians. This society is the most solid guarantee that reform will continue her work in that country. One of its members, M. Moreau (de Tours), after having examined the colony of Gheel, has publicly announced his desire to see a similar colony bestowed upon France. He declares, in his medical letters, that Gheel approaches nearest to the ideal of perfection in the treatment of the insane. The opinion of this savant, who has made a special study of the relative value of all the European establishments, is most precious. He has done us the honour to repeat it in these terms:—"I say it, and repeat it again, what I said fifteen years ago, *there is no asylum that is worth a good colony, and in every country it is possible to colonise the insane.*" In 1840, M. Moreau feared that this establishment, unique in the world, would be destroyed; at the present moment, administrative difficulties might bring about that result; so that the following phrase, borrowed from his letters upon Gheel, is still appropriate:—"If I pronounce myself so openly in favour of the colony; if I endeavour to preserve it from a ruin *that some critics more than severe,* some *unfavourable reports,* have rendered imminent, I am anxious that my sentiments, the nature of the convictions upon which I act, should not be misunderstood. Gheel is, after all, but the imperfect realization of a theoretical idea, for which I preserve all my interest, all my consideration." That also is our opinion. To render Gheel perfect, at least as far as possible, it would be necessary to withdraw the government of the insane from the direction of certain mayors of villages; it would be necessary to concentrate the power in the hands of a chief physician and a director. Nothing was more easy; immense heaths offered every facility for the establishment of an infirmary out of the reach of certain avaricious men, who for many years have trafficked upon the lunatics and their nurses. Thus, a learned man, remarkable for his works and the independence of his mind, asserts that Gheel may be reproduced in every country. Convinced of this truth, he presented to the International Congress of Beneficence, held in 1856, at Brussels, the following proposition:—"To encourage the establishment of colonies of health for the indigent insane, asylums in free air, in which family life should be offered, as the best curative means of insanity." The Congress could not take up a question very secondary amongst those which then waited and still wait for solution, such as that of the means of subsistence, from an economico-political point of view—of pauperism, of emigration, &c. But it received a favourable attention in the section, when it was necessary to vote twice in order to adjourn it to the next Congress. In fact, this question is more important than is imagined; it is a question of the liberation of a considerable number of prisoners, of substituting a productive labour for idleness, to divert public assistance from the erection and maintenance of those immense asylums which England, the United States, and other countries exhibit. We do not despair that soon free colonies, like Gheel, will be established in Germany, France, Russia, and perhaps even in England: nothing is more easy wherever there exist uncultivated lands and sparse populations: the attempt of free colonization will reduce to nothing all the objections which the enemies of this system have heaped together.* Besides, it is easy to understand how persons of good faith are in error, if they admit, without examination, the opinion of the celebrated Esquirol, who, seeing nothing but the necessity of removing the insane from the scene where they had contracted their disease, had said that a lunatic asylum is an instrument of cure, and that in the hands of a skilful physician it is the most powerful therapeutic agent. It is conceivable that isolation in the country, in the midst of a family ordered *ad hoc*, and under the direction of a physician equally skilful, offers much greater advantages.

In France, asylums have assumed forms which no longer resemble those gloomy prisons or hospitals, in which formerly one drew back, in order to take in the horrible spectacle, that of fury and disorder, produced by the amalgamation of all human miseries. At present Bicêtre, La Salpêtrière, Charenton, St. Yon, Maréville, Auxerre, and under the direction of the Ferrus, Parchappe, Baillarger, Moreau, Girard, Renaudin, Calmeil, Lelut, Falret, &c. &c., has become a monument erected to the relief of sick men. We will say further, that many private asylums approach the free establishments, which they imitate as far as possible by the system of family life. Such are the asylums of MM. Voisin and Falret at Vannes; that of M. Brierre de Boismont, a learned psychologist at Paris; that at Passy, under the direction of Dr. Blanche, &c.

In France, as in Belgium; in Germany, as in England, there remain *lacunæ*, that many alienists have pointed out. It would be necessary—

1st. To encourage studies, by creating in schools of medicine, or in the universities, chairs of psychological medicine and cliniques for those diseases.

2nd. To assign the direction of lunatic establishments to especial physicians, and to nominate clinical teachers in the more important public asylums.

3rd. To make it imperative that the public and private establishments should be situated out of towns; that they should be sufficiently extensive to offer to the insane distractions, agricultural works, and gardening; that they should be well kept, and under the responsibility of a physician charged with the hygienic and medical superintendence, as well as with the general direction.

4th. To form country colonies for the insane, possessing a

* These enemies are not all disinterested in the question.

therapeutical centre, under the direction and inspection of an administrative physician.

Why create chairs of psychiatry ?

Because, out of one hundred young physicians, hardly ten will trouble themselves of their own motion about this branch of medicine. Rarely will the remaining nine-tenths be in a position to recognise the development of a mental disease and its origin, although it is often only in that period that cure is possible. Hence proceed the irremediable cases that alienists are called upon to treat at a later stage.

Why make physicians the responsible administrators ?

Why appoint physicians as clinical chiefs in the great establishments ?

Because this is the only means of keeping in the van interests which at first sight appear distinct, but which practice soon recognises to be intimately connected ; interests which the administrator must appreciate in a medical point of view, as well as the clinical chief must do in their applications.

Proved men, like the chief physicians, can and ought certainly to exercise the most absolute authority in a lunatic asylum, in order to possess all the means of action upon mental disease ; and the physician alone is capable of judging of the opportunity for administrative measures relating to the insane.

Why ought all public and private lunatic establishments to be situated in the country ?

Because it is there only that the family life can be realised suitably for the insane, who need air and space to act without danger to any one, and especially to be removed from the circumstances which surrounded the onset of this disease. These establishments would be therapeutical centres, which would have farms as subsidiary establishments. The rich would find these distractions, and the poor would work in the fields.

The air of the fields, as says one of the princes of science, Alexander von Humboldt, is the first and best therapeutical agent. Here are his words, extracted *verbatim* from his celebrated work, *Kosmos* :—"The simple contact of man with nature, that influence of open air—or as other languages have it, in a more beautiful expression, of *free air*—exert a soothing power, they soften *pain*, and allay the *passions*, when the soul is agitated in its utmost depths." This true and noble remark of Humboldt dispenses us from saying another word in favour of colonies, which we maintain to be the holy ark of the cure of insanity.

Who will not be struck with the coincidence of the first reform of the penitentiary system of the insane in France and in England ? It was in 1792 that the celebrated Quaker, Samuel Tuke, was the first to effect a reform in a new asylum established by him near York. The mental disease of George III. had contributed to fix attention on the treatment of insanity. The physicians of the King disputed amongst themselves; discussions took place in the House of Commons and the House of Lords;—all this revealed that psychological studies had made little progress, and that the condition of the insane was most wretched. It is impossible to picture to ourselves the barbarity with which these last were treated.

Later, Parliament instituted several inquiries—in 1815, 1816, and 1827—in consequence of which St. Luke's and Bethlem were ameliorated. At length, England, after having erected admirable asylums in different counties, and exhausted all the modifications of the system of seclusion, about ten years back inaugurated a new system—that of "non-restraint"—invented, it appears, by Dr. Charlesworth, claimed by Dr. Hill, but principally put in practice by Dr. Conolly, formerly chief physician to Hanwell. In this system, the object is not to aggravate the condition of the patient by mechanical restraint. Immediately, satirical attacks were poured forth in France against what appeared *prima facie* an impossibility. Liberty of action, when one is maniacal or demented; union, order, peace, amongst maniacs or melancholics; all detained in spite of themselves in an asylum! It seems, indeed, that the necessities of a closed asylum compel us to certain small violences—to some means of physical or moral control. As to the basis of the question, of what importance is the form of restraint?—be it a barred or a padded cell, handcuffs, or a strait waistcoat—that matters little. But, according to our opinion, the good of this reform must consist in the principle of *gentleness*, which must necessarily be employed. The cares and the patience of which the insane have been the object must also have reacted profoundly on the administration and moral organization of the establishments when this principle is employed. In England, it seems that alienists are still more or less divided as to its value. The Commissioners in Lunacy distributed a circular to all the alienist-physicians, in order to elicit their practical opinion in a report upon the question of the application or rejection of the "non-restraint" system and of "solitary confinement." In conclusion, the Commissioners felt themselves compelled to establish that the suppression of means of restraint was only a question of money—that is to say, that straps and mechanical means of restraint must be replaced by a numerous staff of attendants. They admitted, however, with the greater number of alienists, that cellular seclusion may be necessary during the paroxysms of mania, &c. Dr. Forbes Winslow, the editor and distinguished founder of the *Psychological Journal*, opposes the somewhat too exclusive conclusions of the Commissioners. He thinks that the treatment of an insane person ought, like that of a person not insane, not to depend upon abstract and preconceived ideas; that we must act, according to the medical indications, with conscientiousness, honour, and firmness, without any other consideration than the aim of being useful. We completely approve these ideas; they are quite applicable to the "free-air" system, in which all the necessary precautions are taken to prevent a patient from doing injury to himself or others.

However, it cannot be denied that the principle of sequestration and of medical isolation has sometimes furnished to crime the favourable opportunity of accomplishing its objects over the fortunes, if not over the lives, of those who had been incarcerated. The "free-air" system can never lend itself to this; and this is an immense guarantee. Be it indifference or error, sequestration has ruined a crowd of persons whose nervous dispositions had by this means been rendered altogether insane. A man who might have recovered in a few days in the country, has ended by losing his reason behind the bars of his cell! The treatment of the curable and incurable in the system of seclusion admits but of little difference; it is, at bottom, the penitentiary system of seclusion that rules over the medical treatment. What must we think at the present day, on seeing those endless combinations in order to arrive at cells which resemble the large cages of menageries, and barred beds which resemble the gridiron of St. Lawrence, &c.? All this is invented to subdue, to break the spirit of the patient, who, for the most part, has but too keen a sense of the misfortune that weighs upon him. Honour, then, to England, which has introduced a principle which can only lead to the "Free Air" and the "Family Life." The greater number of those immense asylums, in her bosom and in America, look from afar off like giants destined to swallow up their populations!

In "non-restraint" there remain, it is said, no more straps, straitwaistcoats, cells. So be it; but for the convalescents, the periodical lunatics, peaceful maniacs, the melancholic, the weak-minded, there are ever and always lawns, galleries, and gardens, in which they wander ceaselessly, without finding a friend to guide them in the night of their intelligence. How different from what passes in the families where the lunatic is admitted into ordinary life. The father, the mother, the children, the servants of the house surround him, take care of him, and continually direct his feelings, his affections, his ideas. Compare what passes in the cottage of a peasant, or in the house of a citizen who receives a lunatic at Gheel, were it even a foreigner whose language they could not understand! Behold here medical isolation complete ! It is a society, small indeed, very simple, not versed in all the artificial ceremonies of towns: at the first they must communicate by signs; but there is the beneficent activity of the mind—it is life, in fact. The attention is turned aside from bitter memories and forced to new wants. In the end sympathy is established, and the lunatic may perhaps again be restored to humanity.

The greater number of alienists who advocate the system of seclusion, pretend that the patients have often no wish for communication with the world; they want complete repose of mind in certain cases, and in others the discipline of the cloister. This may be necessary for a brief space; but then how much greater is the peace of mind in the open air, how much more active and more natural is the solitude of the fields, heaths, and springs !

Before concluding, let us say, that free life for the insane has already its defenders and partisans. All the foreign physicians who visited Gheel have expressed their admiration of the establishment, and their regret at seeing that this ancient colony is not yet completely organized; and especially that an infirmary, worthy of the name, and in proportion to the number of the insane, has not yet been constructed, although the law and the regulations which prescribe it are more than *six years* old !

Dr. Droste, of Osnabrück, has developed, in an article in the journal edited by him, the mode of existence of the insane at Gheel, their wants, the absence of organization of the medical service, and the deplorable state of the administration. The rectitude of mind of this writer, his frank generosity, so well known to his numerous readers in Germany, double the value of his approbation. Dr. Moreau de Tours, of whom we have already spoken, approves of the colony in these words:—"Everywhere I have penetrated beneath the roof destined, by public charity or by private speculation for the abode of the insane. I have seen nothing analogous to Gheel."

In Italy, Dr. Biffi, of Milan, declares that this colony is destined to execute on a large scale a great reform in the treatment of the most terrible of maladies; and that it would be folly to think that this could be accomplished without organizing there a medical body capable of leading the way in this important attempt. *It is this experience that science and humanity claim from so advanced a nation as Belgium.*

Dr. John Webster, of London, who has, more than any other physician, engaged himself in describing the asylums of the Continent, was much pleased with his visit to Gheel; and his opinion too will shortly be published in the *Psychological Journal.*

Lastly, Dr. Willis directs, as it appears, a "Free Air" asylum at Great Ford, in England. Dr. Jessen directs a similar house at Hornheim, in Holstein: the patients even go to execute errands, and walk in Kiel. MM. Falret and Voisin possess one at Vanvres, near Paris, which approaches nearly to what we may regard as belonging to the system whose excellence we have attempted to describe.

In writing these lines we have discharged a duty of conscience. It is possible that error may have slipped in involuntarily under our pen; but never can that pen describe the torments that we have witnessed, and yet exist, in the cells and yards so strongly as they have been felt!

To resume. We believe that civilization advances only when egoism and falsehood retreat in the social world. This movement is evidently going on; if not in all countries, at least here and there. And this progress cannot be better measured than in the development of duty in the public conscience, especially towards the infirm and the insane, who might be deceived almost with impunity, if the moral sense did not everywhere interdict. The insane, then, are the dumb victims of our unworthiness, or the living witnesses of the degree of our true civilization.

---

